# A Comparative Assessment of Primary Implant Stability Using Osseodensification vs. Conventional Drilling Methods: A Systematic Review

**DOI:** 10.7759/cureus.46841

**Published:** 2023-10-11

**Authors:** Abdulrahman K Althobaiti, Abdulrahman W Ashour, Firas A Halteet, Sulaiman I Alghamdi, Mohamed M AboShetaih, Ali Mosa Al-Hayazi, Ahmed M Saaduddin

**Affiliations:** 1 Dentistry Program, Batterjee Medical College, Jeddah, SAU; 2 Oral and Maxillofacial Surgery, Dental Sector, Ministry of Health, Dakahlia Governorate, Mansoura, EGY; 3 Dentistry, Suliman Habter Dental Centers, Abha, SAU; 4 Oral and Maxillofacial Surgery Department, Faculty of Dentistry, Mansoura University, Mansoura, EGY; 5 Division of Oral and Maxillofacial Surgery, Department of Clinical Dental Sciences, Dentistry Program, Batterjee Medical College, Jeddah, SAU

**Keywords:** primary implant stability, isq, insertion torque, osseointegration, bone density, resonance frequency analysis, osseodensification, implant stability quotient, densah bur, conventional drilling

## Abstract

Osseodensification is a novel biomechanical bone preparation technique that has been established to replace conventional bone drilling and therefore will optimize the implant site. The purpose of this systematic review was to compare the implant stability obtained by osseodensification drilling to those associated with conventional drilling techniques. An electronic search was performed in the PubMed, Scopus, EMBASE, Cochrane Oral Health Group, and Dentistry and Oral Science Source databases searched through Elton B. Stephens Company (EBSCO) for potentially relevant publications in the English language from January 2013 to December 2022. Randomized clinical trials (RCTs) and non-randomized studies of interventions (NRSIs), contrasting osseodensification drilling with conventional drilling, studies documenting implant stability quotient (ISQ), and studies reporting the immediate outcome and at least three months of follow-up after dental implant placement were included. Two independent investigators evaluated the quality of the reviewed studies to determine the risk of bias using the version 2 of Cochrane risk-of-bias (RoB) tool for RCTs (RoB 2) and RoB for NRSIs (ROBINS-I). Majority of the studies showed that bone density was significantly higher in the osseodensification group. The overall RoB for the NRSIs was reported to be low with respect to confounding, selection, classification, incomplete data, deviance from interventions, outcome evaluation, and selective reporting. The quality assessment of the RCT studies included in the review using the RoB 2 tool showed a high overall risk. The findings of the current review reveal that osseodensification drilling exhibited higher resonance frequency analysis (RFA) and ISQ values than conventional drilling protocols. Similarly, when osseodensification regions were contrasted with traditional drilling, bone density at the implant surface was augmented.

## Introduction and background

Osseodensification is an innovative biomechanical method for bone preparation, designed to supplant traditional bone subtractive drilling, ultimately enhancing the quality of the implant site [1]. It intends to induce a compression movement at the contact point of an osseous drill with a specifically made bur termed a Densah bur, resulting in controlled osseous deformation due to the intrinsic nature of skeletal tissue viscoelasticity and viscoplasticity. When compared to the conventional subtractive drilling technique, this method improves the primary and secondary stability of the implant and the percentage of bone-implant contact (BIC) by up to threefold. The main benefits of this strategy are the conservation of the bone volume, accelerated healing due to the protection of the bone matrix, and consistent replacement of the autogenous bone graft matrix along the implant surface [2]. In contrast to conventional drilling protocols, osseodensification improves primary stability by centrifugally densifying the drilled osteotomy site using non-subtractive drilling [3].

Primary implant stability is proffered by frictional forces between the implant surface and the periosteal walls of the osteotomy location. This technique is also associated with greater insertion and removal torque and bone volume around implants. Osseointegration is a phenomenon that represents the functional contact between natural bone and implant resulting in new bone formations on the implant surface, enabling it to accomplish secondary stability [4,5]. Conventional drilling employs a positive rake angle to retrieve a thin layer of tissue with each flute pass, resulting in an osteotomy without any bone residuals. The osseodensification, on the other hand, employs four slender flutes at a negative rake angle to generate a layer of compact, dense bone enclosing the wall of the osteotomy. The bone expands at a controlled predetermined rate, and the densifying bur could indeed cut in either the anti-clockwise or clockwise direction^ ^[6,7].

The type and density of the bone, surgical guidelines, implant thread type, geometrical configuration, and surface design of the implant are important factors in fostering implant primary stability [8].The insertion torque (IT) peak was found to be proportional to implant primary stability and bone density. High IT may cause a significant increase in the initial bone-implant contact ratio (BIC%) [1,5]. IT and resonance frequency analysis (RFA) has been demonstrated to be objective indicators of bone density and to have a positive association with initial implant stability [9,10].^ ^Primary stability is obtained when implant micromotion is confined to less than 50- to 150-µm thresholds until osseointegration begins [11]. While the osseodensification drilling process has been evidenced in benchtop in vitro and animal studies, the direct measurement of its biomechanical merits in clinical studies is lacking, which would indeed be of great interest [4,6,7].^ ^Henceforth, the purpose of this systematic review was to make comparisons of the implant stability obtained by osseodensification drilling to those associated with conventional drilling techniques.

## Review

Methodology

*Protocol* 

The Preferred Reporting Items for Systematic Reviews and Meta-analyses (PRISMA) guideline was applied to structure the systematic review based on the patient, intervention, comparison, outcome, and study design (PICOS) criteria [12].

Research Question

The addressed research question was “Does the implant stability (O) differ between osseodensification drilling (I) and the conventional drilling technique (C) in individuals receiving implant placement (P) under a controlled clinical setting (S)?"

*Eligibility Criteria* 

Randomized clinical trials (RCTs) and non-randomized studies of interventions (NRSIs), studies contrasting osseodensification drilling with conventional drilling, studies documenting implant stability quotient (ISQ), and studies reporting post-surgical findings immediately and at least 3 months of follow-up upon placing dental implants were included. Non-intervention studies, case reports, in vitro studies, animal studies, studies not providing information on osseodensification drilling, studies lacking a conventional control group or not reporting the conventional drilling system, and studies on patients receiving radiation therapy of the head and neck or with systemic disease states were excluded.

Search Strategy

An electronic search was performed in the PubMed, Scopus, EMBASE, Cochrane Oral Health Group, and Dentistry and Oral Science Source databases searched through Elton B. Stephens Company (EBSCO) for potentially relevant publications in the English language from January 2013 to December 2022. Medical Subject Headings (MeSH) terms were used as follows: ("Osseodensification" OR "Densification" OR "Osseointegration" OR "Conventional Drilling" OR "Densah bur" OR "Implant Osteotomy") AND ("Dental implants" OR " Implant stability" OR "Bone-implant contact" OR "Implant stability quotient" OR "Bone density") AND ("Prospective" OR "Randomized Controlled Clinical Trials"). A manual search was also conducted on the reference lists of the selected articles that were not obtained in the electronic database search results. Articles not written in English, duplicate records, and studies that were not centered on osseodensification were not considered further.

Study Selection, Data Collection, and Data Extraction

**Figure 1:**
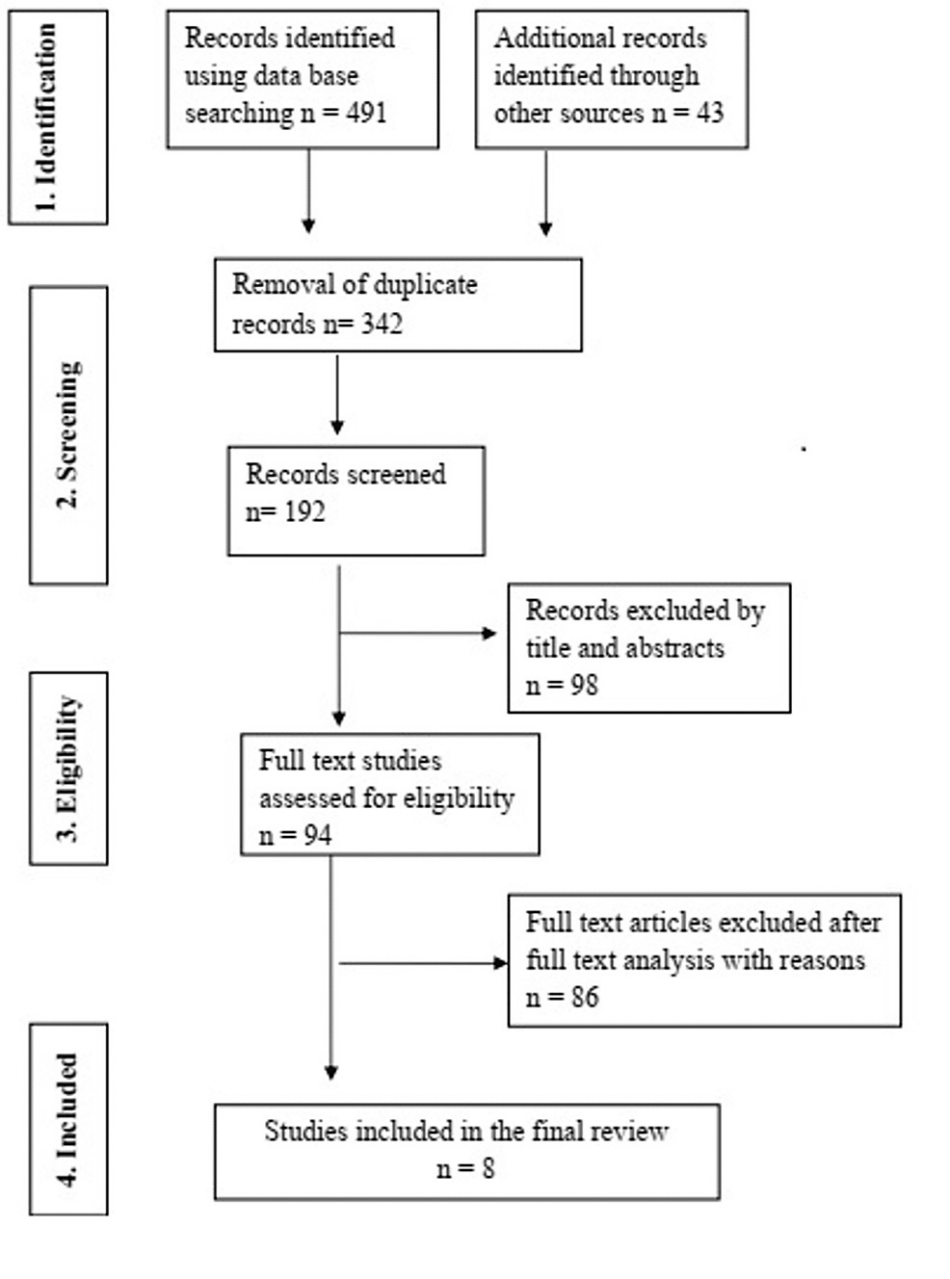
Preferred Reporting Items for Systematic Reviews and Meta-analyses (PRISMA) flow chart of the included studies

Studies were evaluated based on their title or abstract, and those that fulfilled the inclusion criteria were chosen for full-text review. Two authors evaluated the studies that would be included, and in the event of disagreement, the third author was consulted. Figure 1 depicts the search and selection method. A predetermined table was used to collect the name of the author, publication year, study design, number of participants, study groups, implant types, outcome measures, and findings from the studies reviewed. In the event of a discrepancy in the information extracted, the corresponding authors of the respective article were summoned.

Quality Assessment

Two independent investigators evaluated the quality of the RCTs using the Cochrane Collaboration tool for determining the risk of bias (RoB) [13]. The tool confronts seven distinct domains, namely, sequence generation, allocation concealment, blinding of participants and personnel, blinding of outcome assessment, incomplete outcome data, selective outcome reporting, and other issues. The investigators assigned an estimated RoB for each of the included studies. When all of the criteria were met, the RoB was low. If any of these criteria were lacking, the RoB was moderate. When two or more criteria were missing, the RoB was considered high.

The seven domains of ROBINS-I (RoB in NRSIs) were utilized to assess the quality of studies. The first two domains are confounding and selection bias, which considers factors prior to the start of the interventions as preliminary data. The third domain is associated with intervention categorization. The four remaining domains recognize issues that emerge after the start of interventions, such as biases induced by deviations from envisioned interventions, incomplete or missing information, outcome assessment, and selective reporting of results. The RoB was classified as low, moderate, serious, critical, or no information [14].

The electronic search yielded 491 articles, and the hand search yielded 43 studies (Figure 1). There were 192 articles left after duplicates were eliminated. Following a review of the title and abstract, 98 records were removed. Out of 94 articles that qualified for full-text screening, 86 studies were exempted since they lacked a control group that did not contrast the conventional drilling or contained insufficient data. Henceforth, the qualitative synthesis contained eight clinical studies [15-22].

Results

Selection of Studies

There were seven split-mouth RCT studies [15-21] and one NRSI study [22]. Table 1 summarizes the study methods, outcome measures, and implications of these studies.

**Table 1:** Characteristics of the included studies in the review (methods, outcome measures, and implications) RCT: randomized clinical trial; ISQ: implant stability quotient; RFA: resonance frequency analysis; SLA: sandblasted and acid-etched; HU: Hounsfield units; IT: insertion torque; CBCT: cone-beam computed tomography; NRSI: non-randomized studies of intervention

Author, year	Study design	Sample size	Control group	Test group	Implant type	Follow-up	Measured outcome and clinical findings
Aboelnaga et al., 2019 [15]	RCT	20 patients, 20 implants	Ridge expansion using conventional drilling	Osseodensification sinus lift Densah bur (anti-clockwise rotation)	T6 bone level implant by NucleOSS between 3.5 and 4.8 mm in diameter	Baseline, 3 and 6 months	The test group disclosed a significantly higher ISQ using RFA. The bone density values (HU units) were also found to have a significant difference between the groups.
Al Ahmari, 2022 [16]	Split mouth	20 patients, 40 implants	Conventional drilling	Osseodensification Densah burs	NS	Baseline, 7 and 12 months	The osseodensification technique enhances better primary stability in the low-density bone regions, augments the healing process, and thereby maintains marginal bone integrity and bone density.
Aloorker et al., 2022 [17]	Split mouth	10 patients, 20 implants	Conventional drilling of the right maxillary posterior region	Osseodensification (anti-clockwise rotation) of the left maxillary posterior region	Threaded SLA implants	Baseline, 3 and 6 months	The bone density and crestal bone level were measured using the Planmeca Romexis software. The bone density (HU units) was significantly increased in the test group and remained relatively dense over a period of 6 months, providing a primary and secondary stability.
Arafat and Elbaz, 2019 [18]	RCT	24 patients, 24 implants	Osteotome sinus lift in the partially atrophic maxilla	Osseodensification sinus lift in the atrophic maxilla using Densah bur (anti-clockwise rotation)	Tapered screw implant	Baseline, 6 and 12 months	RFA was used to measure the ISQ. The test groups showed a statistically highly significant increase in primary and secondary implant stability.
Bergamo et al., 2020 [19]	Multicenter prospective trial	56 patients, 150 implants	Conventional drilling in the mandibular posterior and maxillary anterior or posterior region	Osseodensification in an equal distribution of arch, area operated, and implant dimensions as the control group	Narrow-, regular-, or wide-diameter and short-, regular-, or long-length implants using strong SW Plus (S.I.N Implant System, SaoPaulo), Zimmer Biomet (Warsaw, IN), and IS-III Active (NeoBiotech, CA)	Baseline, 3 and 6 weeks	Higher IT and ISQ (RFA) were found in the test group with the exception of short implants.
Hassan et al., 2021 [20]	Split mouth	7 patients, 14 implants	Conventional drilling in the right maxillary region	Osseodensification Densah bur kit (clockwise and anti-clockwise rotation) of the left maxillary region	NS	Baseline, 6, 7, and 12 months	CBCT showed improved bone density in the test group that worked safely in the low-density bone with rapid healing and reduced chance of creating bone dehiscence. ISQ was found to be insignificant.
Ibrahim et al., 2020 [21]	Split-mouth RCT	10 patients, 20 implants	Conventional drilling maxillary	Osseodensification Densah bur (clockwise rotation)	Dentium two-piece implant (10 mm)	Baseline, 4 months	ISQ using RFA was used to determine the amount of implant stability.
Sultana et al., 2020 [22]	NRSI	20 patients, 20 implants	Conventional drilling of the anterior maxilla	Osseodensification Densah bur kit (clockwise and anti-clockwise rotation)	ADIN Touareg S spiral dental implants of various diameters and lengths	Baseline, 6 and 8 months	The stability of the implants was measured using RFA. Crestal bone levels and ISQ were found to be statistically insignificant between the groups.

Characteristics of the Selected Studies

Four of the included studies were from Egypt [15,18,20,21], one from Saudi Arabia [16], and two from India [17,22]. One study was a multicenter controlled clinical trial that took place in Brazil, the United States, and Chile [19]. All implants were placed in the maxilla using the osseodensification technique and evaluated by comparing them with conventional drilling.

Aboelnaga et al. [15] used 20 implants that were assigned randomly to ridge expansion or osseodensification groups. ISQ values were obtained through RFA, and bone density values were ascertained through a postoperative cone-beam computed tomography (CBCT) scan in Hounsfield units. After six months of implant placement, the osseodensification patients had a higher mean bone density of 670.10±56.20 HU than the ridge expansion group (525.95±74.89 HU). The mean RFA readings of the bone expander and osseodensification groups was 69.30 ± 2.58 ISQ and 86.40±3.50 ISQ, respectively (p<0.01) [15].

Al Ahmari [16] used CBCT to draw comparisons between the osseodensification drilling technique and the conventional approach in terms of primary implant stability and other factors, such as plaque index, bleeding on probing, pocket depth, and radiographic evaluation of the bone density and marginal bone loss. The split-mouth study utilized 40 implants in total, and both clinical and radiographic assessments were accomplished immediately after surgery, as well as at seven and 12 months. While bone density was found to be statistically significant in the osseodensification group, other variables in the study were found to be insignificant.

Aloorker et al. [17] conducted a split-mouth study consisting of 10 patients each in the osseodensification and conventional groups in the maxillary posterior region. CBCT bone density and crestal bone levels were determined three months after implant placement and were found to be significantly increased after osseodensification over six months, facilitating primary stability and osseointegration.

Arafat and Elbaz [18] included 24 patients who desired one to two implants in the posterior maxilla and had at least 5 mm of residual bone height in two groups: conventional osteotome strategy to uplift the sinus membrane and osseodensification for crestal sinus elevation [18]. RFA was used to ascertain primary stability after implant placement and secondary stability was evaluated six months later. The difference in the bone height and ISQ values between the groups was found to be statistically significant (p=0.001). In the osteotome and osseodensification groups, the increase in bone height (bone gain) was 2.79±0.30 mm and 3.33±0.25 mm, respectively. Despite the significant increase in the ISQ values in both groups from baseline (immediately after implant placement) and at six months postoperatively (p≤0.001), the osseodensification group showed a significantly higher ISQ values at both evaluation time intervals [18].

Bergamo et al. [19] studied 150 implants in a multicenter-controlled clinical trial. Patients received treatment with narrow, regular, or wide implants and short, regular, or long implants in the anterior or posterior maxilla/mandible. IT and ISQ were measured respectively using torque indicator and RFA immediately after surgery, three and six weeks later. With the exception of short implants, higher IT and ISQ values in the osseodensification group were found, regardless of arch or areas operated, and implant design and geometry [19].

Hassan et al. [20] used 14 implants in a split-mouth design that were clinically and radiographically monitored with CBCT for 12 months. It was demonstrated that the osseodensification approach promotes the healing process and preserves the marginal bone integrity after implant placement. The ISQ of osseodensification and the conventional group was determined to be statistically insignificant. The comparison of bone densities showed a statistically significant difference in the pursuit of the osseodensification group at the baseline and an insignificant difference at 7- and 12-month post-implant placement [20].

Ibrahim et al. [21] used a split-mouth design to place 20 implants in 10 patients who had at least two missing teeth in the maxillary posterior region. CBCT was performed immediately following the procedure and yet again six months later to determine the extent of ridge expansion, marginal bone level, and alteration in bone density surrounding implants. Implant stability was determined immediately after implant placement using RFA with 74.25±4.95 ISQ vs. 59.65±5.39 ISQ and four months later with 76.90 ±4.05 ISQ vs. 68.25 ±5.14 ISQ [21].

Sultana et al. [22] included 20 patients and divided them into two groups using the conventional and osseodensification drilling techniques. Primary stability was assessed in both groups using Osstell RFA at baseline and after six months, whereas the crestal bone levels were assessed at baseline and six and eight months postoperatively. In terms of crestal bone levels, there was a statistical insignificance difference (p > 0.05) between the groups. The primary stability of the implant, nevertheless, was found to be relatively higher in the osseodensification group [22].

RoB Within Studies

The overall RoB for the NRSI conducted by Sultana et al. [22] was reported to be low with respect to confounding, selection, classification, incomplete data, deviance from interventions, outcome evaluation, and selective reporting (Table 2). On the contrary, the RCTs reviewed had a high RoB in the context of allocation concealment and blinding (Table 3). Only Bergamo et al. [19] blinded the participants and the investigator. Despite the fact that the studies were of split-mouth design, randomization was not mentioned in four of the included studies [16,17,19,20].

**Table 2:** Quality assessment of the NRSI included in the review using the RoBINS-I tool RoBINS-I: risk-of-bias tool for non-randomized studies of interventions; RoB: risk of bias

Author year	Pre-intervention phase		Intervention phase	Post-intervention phase				Overall risk
	Confounding bias	Selection bias	Misclassification bias	Performance bias	Attrition bias	Detection bias	Reporting bias	
Sultana et al., 2020 [22]	Low RoB	Low RoB	Low RoB	Low RoB	Low RoB	Low RoB	Low RoB	Low RoB

**Table 3:** Quality assessment of the randomized controlled studies included in the review using the RoB 2 tool RoB: risk of bias; RoB 2: version 2 of Cochrane risk-of-bias tool for randomized controlled trials

Author, year	Selection bias		Performance bias	Detection bias	Attrition bias	Reporting bias	Other biases	Overall risk
	Random sequence generation	Allocation concealment	Blinding of participants and investigator	Blinding of outcome assessment	Incomplete outcome data	Selective reporting		
Aboelnaga et al., 2019 [15]	Low RoB	High RoB	High RoB	Low RoB	Low RoB	Low RoB	Uncertain RoB	High RoB
Al Ahmari. 2022 [16]	Uncertain RoB	High RoB	High RoB	Low RoB	Low RoB	Low RoB	Uncertain RoB	High RoB
Aloorker et al., 2022 [17]	Uncertain RoB	High RoB	High RoB	Low RoB	Low RoB	Low RoB	Uncertain RoB	High RoB
Arafat and Elbaz. 2019 [18]	Low RoB	High RoB	High RoB	Low RoB	Low RoB	Low RoB	Uncertain RoB	High RoB
Bergamo et al., 2020 [19]	Uncertain RoB	High RoB	Low RoB	Low RoB	Low RoB	Low RoB	Uncertain RoB	High RoB
Hassan et al., 2021 [20]	Uncertain RoB	High RoB	High RoB	Low RoB	Low RoB	Low RoB	Uncertain RoB	High RoB
Ibrahim et al., 2020 [21]	Low RoB	High RoB	High RoB	Low RoB	Low RoB	Low RoB	Uncertain RoB	High RoB

Discussion

The long-term clinical outcomes of implants utilizing osseodensification hold significant clinical importance. Understanding these outcomes can provide valuable insights into the effectiveness and reliability of this technique in ensuring the stability and success of dental implants over extended periods. Such data can help clinicians make informed decisions about the use of osseodensification in implant procedures, ultimately benefiting patient care and treatment outcomes. The current systematic review established a clinical comparison of the outcomes of RCT and NRSI studies, which proficiently exhibited increased ISQ values at baseline and follow-up for osseodensification when contrasted with conventional drilling techniques.

According to the findings of this systematic review, the potentially positive effect of utilizing osseodensification for osteotomy preparation is bone compaction autografting. The anti-clockwise rotation of the bur pushes retained autogenous bone fragments apically and laterally. This autogenous compacted graft within the osteotomy not only offers additional mechanical primary stability against the implant but can also act as a nucleating agent for pivotal new bone growth around the implant. This improves overall implant stability during the early healing stage [23]. Several previous systematic reviews focused solely on animal studies [1] or collective evidence from animal models and human clinical studies [9,24-26]. However, Gaspar et al. [27] analyzed data entirely from human subjects, which is compatible with the findings of the current review.

Contemporary histologic findings from animal studies signify that osseodensification increases bone-implant contact (BIC) and bone-area fraction [5,7,10,11,28-32]. Neiva et al. [33] conducted human clinical trials and found that osseodensification crestal sinus floor elevation with chemically synthesized and biodegradable calcium phosphosilicate putty produced favorable and predictable results. Koutouzis et al. [23] and Huwais et al. [34] carried out retrospective multicenter research of 28 implants for ridge expansion and 261 implants for crestal sinus elevation with six-week and five-year follow-up periods, respectively. Similarly, Gaspar et al. [27] performed a prospective study with 97 implants to evaluate the effect of osseodensification for ridge expansion, crestal approach sinus elevation, immediate implant placement, and full-arch situations with immediate loading and found effective results for bone expansion and mitigating peri-implant bone fenestrations or dehiscences.

There are various methods for determining implant primary stability. They are divided into two categories: invasive and non-invasive methods. IT is an estimate of the rotational friction of the implant and is a good predictor of primary implant stability. RFA is primarily focused on the resonance frequency of the implant-bone complex at the time of implant placement. This method is perhaps costly and skill-dependent [35,36]. The frequency response of the device was monitored by connecting a transducer to the implant in the buccolingual direction. The resonant sign was calibrated at frequencies ranging from 5 to 15 kHz, with a maximal amplitude of 1 V, and the initial flexural resonant frequency was recorded [22].

The drilling method is yet another important consideration when primary stability is anticipated to be established expediently. Several treatment methods have been compiled with the goal of increasing primary stability, notably in low-density bone. Nevertheless, they all make a comparison of subtractive drilling performed under the supposition that bone must therefore be removed from the site. It was further documented that with varying degrees of under-preparation of the osteotomy, enhanced stability can be accomplished. In broader terms, increasing implant diameters combined with smaller osteotomy measurements contribute to significantly greater IT levels during implant placement [23].

The osseodensification drilling technique, on the other hand, is rooted in the idea of non-subtractive multi-step drilling, such as through burs that allow bone preservation and autografting compaction along the osteotomy wall. The densifying bur has a snipping chisel and a tapered shank that allows it to eventually increase the diameter as it moves deeper into the osteotomy region, influencing the expansion. Furthermore, at elevated drilling speeds, drilling can be performed across both clockwise and anti-clockwise rotations. The anti-clockwise drilling orientation is more effective at densification and is employed in low-density bones, whereas the clockwise drilling is used in higher-density bones [8,37]. 

In a novel osseodensification strategy devised by Rodda et al. [38], an innovative approach was employed using Densah burs characterized by multiple grooves and an increasing diameter in an anti-clockwise direction. This design was strategically intended to optimize the preparation of the implant site, concurrently augmenting the stability of the implant upon insertion. The distinguishing feature of this method lies in the counterclockwise rotation of these burs, leading to a conjecture of autogenous bone compaction at the apical extremity. This intriguing hypothesis paves the way for a gentle elevation of the sinus membrane, rendering the technique particularly valuable for sinus lifts. Remarkably, the utilization of osseodensification burs in this context obviates the necessity for graft materials post-sinus augmentation, rendering it a minimally invasive procedure with promising clinical implications.

The osseodensification technique was contended to have enhanced IT from 25 Ncm for implants placed using the standard drilling technique to 49 Ncm in low-density bones. The diameter of the osseodensified osteotomy location was reduced by 91% if it continues to remain empty [37]. This was mainly ascribed to the viscoelastic characteristic of the bone, and it was deduced that the viscoelasticity induces the bone to bounce up, generating a compressive load against the implant. Because of the presence of a considerably large amount of cancellous bone, osseodensification may be especially effective during implant insertions in the maxillary arches. Due to the scarcity of available data for the mandibular region, it should be utilized with prudence in predominantly cortical or denser bone, such as the mandibular anterior region. Furthermore, osseodensification drills have been reported to raise the temperature and may cause tissue damage to surrounding osteoblasts if not applied in conjunction with profuse irrigation [25].

The follow-up durations of the reviewed studies were not normalized, which could demonstrate the heterogeneity of the findings of the review. Moreover, as the current study could only focus on providing estimates for ISQ values, it would be essential to expand toward other clinical features in the future. However, well-designed prospective cohort studies and randomized controlled clinical trials on human subjects are warranted to completely define the biologic dimension and clinical effects of the osseodensification technique.

## Conclusions

The current review reveals higher implant stability and improved bone density for implants installed with the osseodensification drilling method when compared with the conventional drilling protocols, as shown with the RFA and ISQ values and the Hounsfield units in radiographic assessments. These findings can be attributed to the improved bone healing process due to osseous tissue preservation and local autografted bone matrix along the osteotomy bed associated with the osseodensification drilling approach. Researchers and clinicians should closely examine these outcomes to evaluate the effectiveness and long-term success of osseodensification as a bone preparation technique for dental implant placement. This information aids in informed decision-making and contributes to the ongoing refinement of dental implant procedures, ultimately benefiting patient care and treatment outcomes.
